# Microbial communities are thermally more sensitive in warm-climate lizards compared with their cold-climate counterparts

**DOI:** 10.3389/fmicb.2024.1374209

**Published:** 2024-04-15

**Authors:** Xia-Ming Zhu, Jun-Qiong Chen, Yu Du, Chi-Xian Lin, Yan-Fu Qu, Long-Hui Lin, Xiang Ji

**Affiliations:** ^1^College of Life Sciences, Nanjing Normal University, Nanjing, China; ^2^Zhejiang Provincial Key Laboratory for Water Environment and Marine Biological Resources Protection, College of Life and Environmental Sciences, Wenzhou University, Wenzhou, China; ^3^Hainan Key Laboratory of Herpetological Research, College of Fisheries and Life Sciences, Hainan Tropical Ocean University, Sanya, China; ^4^Herpetological Research Center, College of Life and Environmental Sciences, Hangzhou Normal University, Hangzhou, China

**Keywords:** 16S rRNA gene sequencing, cold-climate lizard, fecal and small-intestinal microbiota, thermal adaptation, warm-climate lizard

## Abstract

Environmental temperature affects the composition, structure, and function of the gut microbial communities in host animals. To elucidate the role of gut microbiota in thermal adaptation, we designed a 2 species × 3 temperatures experiment, whereby we acclimated adult males of two agamid lizard species (warm-climate *Leiolepis reevesii* and cold-climate *Phrynocephalus przewalskii*) to 20, 28, and 36°C for 2 weeks and then collected their fecal and small-intestinal samples to analyze and compare the microbiota using 16S rRNA gene amplicon sequencing technology. The fecal microbiota displayed more pronounced interspecific differences in microbial community than the small-intestinal microbiota in the two species occurring in thermally different regions. The response of fecal and small-intestinal microbiota to temperature increase or decrease differed between the two species, with more bacterial taxa affected by acclimation temperature in *L. reevesii* than in *P. przewalskii.* Both species, the warm-climate species in particular, could cope with temperature change by adjusting the relative abundance of functional categories associated with metabolism and environmental information processing. Functional genes associated with carbohydrate metabolism were enhanced in *P. przewalskii*, suggesting the contribution of the fecal microbiota to cold-climate adaptation in *P. przewalskii*. Taken together, our results validate the two hypotheses tested, of which one suggests that the gut microbiota should help lizards adapt to thermal environments in which they live, and the other suggests that microbial communities should be thermally more sensitive in warm-climate lizards than in cold-climate lizards.

## Introduction

The co-evolution of a host and its microbes colonizing different parts of the body including the body surface, oral cavity, gastrointestinal tract, and genitourinary tract is determined by complex symbiotic relationships (Ley et al., 2008; Chevalier et al., 2015). Numerous internal and external factors potentially affect the microbial community composition and structure, but the effects of temperature are more important and pervasive compared to many other external factors (Kohl and Yahn, 2016; Fontaine et al., 2018; Moeller et al., 2020; Zhu et al., 2021; Du et al., 2022; Huang et al., 2022). For example, warming decreases the relative abundance of Firmicutes in the gut microbiome and increases the ratio of putative pathogens in the western fence lizard *Sceloporus occidentalis* (Moeller et al., 2020) and alters the composition of gut microbial communities in the red-backed salamander *Plethodon cinereus* (Fontaine et al., 2018). The reduced relative abundance of bacteria of the genera *Cellvibrio* and *Stenotrophomonas* may adversely influence digestive performance in *P. cinereus* (Fontaine et al., 2018). Other studies on the brown bear *Ursus arctos* (Sommer et al., 2016), the Mongolian gerbil *Meriones unguiculatus* (Khakisahneh et al., 2020), and the thirteen-lined ground squirrel *Ictidomys tridecemlineatus* (Regan et al., 2022) provide indirect and direct evidence for the role of the microbiota in host physiology and survival. For example, compared with germ-free mice colonized with the gut microbiota from hibernating brown bears, germ-free mice colonized with the gut microbiota from summer brown bears trigger obesity, suggesting that seasonal changes in the gut microbiota can affect host energy metabolism and thereby result in mass gains of the body and fat (Sommer et al., 2016). Another example from *I. tridecemlineatus* shows that gut ureolytic microbes convert urea nitrogen into metabolites that can be absorbed by the host to promote protein balance during hibernation (Regan et al., 2022).

Host animals used to examine the effect of temperature on gut microbiota are either from the field, or maintained under controlled conditions in the laboratory. Field studies have examined the effects of external factors including seasonal temperature fluctuations on gut microbiota in a diverse array of vertebrate taxa hibernating [e.g., the brown frog *Rana dybowskii* (Tong et al., 2020), the Chinese alligator *Alligator sinensis* (Tang K.-Y. et al., 2019), *I. tridecemlineatus* (Chiang et al., 2022), *U. arctos* (Sommer et al., 2016), the greater horseshoe bat *Rhinolophus ferrumequinum* (Xiao et al., 2019), and the least horseshoe bat *Rhinolophus pusillus* (Liu et al., 2023)], or not hibernating [e.g., the plateau pika *Ochotona curzoniae* and the yak *Bos mutus* (Fu et al., 2020)] in winter. These field studies have yielded many interesting findings. In *A. sinensis*, for example, the increased relative abundance of bacteria of the genus *Bacteroides* in winter promotes the degradation of host mucin glycans, which is beneficial for hibernating Chinese alligators to pass the winter fasting period (Tang K.-Y. et al., 2019). Studies conducted under controlled conditions in the laboratory have paid more attention to the effect of temperature on gut microbiota. These laboratory studies consistently show that temperature increase or decrease affects the microbial community composition and structure, but conclusions are often drawn based on single species from a single sampling locality (e.g., Khakisahneh et al., 2020; Moeller et al., 2020; Arango et al., 2021; Hassenrück et al., 2021; Zhu et al., 2021; see also Chen et al., 2022). Whether and how the responses of gut microbiota to temperature change differ between host species distributed in thermally different regions or using thermally different habitats remain a sparsely studied area, but data on this topic are of great value in elucidating the role of gut microbiota in thermal adaptation. For example, gut microbial communities in tadpoles are more plastic in the invasive American bullfrog *Lithobates catesbeianus* than in the non-invasive green frog *Lithobates clamitans*, making the former species better able to adapt to a changing thermal environment (Fontaine and Kohl, 2020). Further work could usefully conduct comparative studies on multiple species from thermally contrasting environments to determine the role of gut microbiota in thermal adaptation.

**FIGURE 1:**
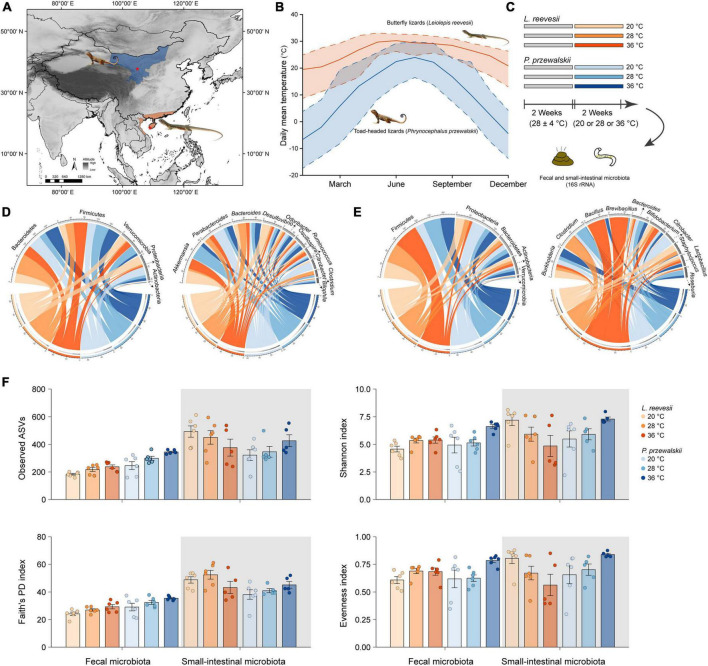
**(A)** Sampling localities (asterisks) of two agamid lizard species, the Reeves’ butterfly lizard *Leiolepis reevesii* having a warm-climate distribution (orange) and the Przewalski’s toad-headed lizard *Phrynocephalus przewalskii* having a cold-climate distribution (blue). **(B)** Daily mean (solid line), maximum (upper dashed line) and minimum (lower dashed line) temperatures of the two sampling localities during 2010–2019 (http://www.cma.gov.cn). **(C)** A 2 species × 3 acclimation temperatures experiment testing the hypotheses associated with the role of gut microbiota in thermal adaptation. **(D)** The relative abundance of the fecal microbiota at the phylum and genus levels in *L. reevesii* and *P. przewalskii* acclimated to three temperatures (20, 28, and 36°C) for 2 weeks. **(E)** The relative abundance of the small-intestinal microbiota at the phylum and genus levels in *L. reevesii* and *P. przewalskii* acclimated to three temperatures (20, 28, and 36°C) for 2 weeks. **(F)** Alpha diversity matrices of microbial communities in *L. reevesii* and *P. przewalskii*.

Here, we focused on two oviparous lizard species of the family Agamidae, the Reeves’ butterfly lizard *Leiolepis reevesii* and the Przewalski’s toad-headed lizard *Phrynocephalus przewalskii*, living in thermally contrasting environments. *L. reevesii* is a medium-sized (up to 166 mm snout vent length, SVL), warm-climate lizard occurring in South China (Guangdong, Guangxi, and Hainan) and Vietnam (Figure 1A; Du et al., 2011), where air temperatures vary from 7.0 to 35.5°C.^1^ *P. przewalskii* is a small (up to 66 mm SVL), cold-climate lizard occurring in North and Northwest China (Gansu, Inner Mongolia, Ningxia, and Qinghai) (Figure 1A; Wang, 2011), where air temperatures vary from −24.8 to 38.9°C (see text footnote 1). We designed a 2 species × 3 temperatures experiment, whereby we acclimated adult males of the two lizard species to 20, 28, and 36°C for 2 weeks and then collected their fecal and small-intestinal samples to analyze and compare the microbiota using 16S rRNA gene amplicon sequencing technology. We tested the following two hypotheses. First, the gut microbiota should help lizards adapt to thermal environments in which they live and, as such, gut microbial genes related to metabolic function should differ between the cold- and warm-climate species. Second, gut microbial communities should be more sensitive to temperature change in the warm-climate species living in thermally more stable environments.

## Materials and methods

### Animal collection, experimental design, and sample collection

In mid-July 2020, we collected adult males of *L. reevesii* (103–152 mm SVL; 25.4–37.5 g body mass) from Ledong (18°29′N, 108°57′E), Hainan, South China, and adult males of *P. przewalskii* (52–61 mm SVL; 4.7–7.6 g body mass) from Zhongwei (37°27′N, 104°52′E), Ningxia, Northwest China (Figure 1A). Daily mean, maximum and minimum temperatures are higher in Ledong than in Zhongwei throughout the year (Figure 1B). All lizards were transferred to our laboratory in Sanya, Hainan, where four individuals of the same species were housed together in each 1.2 × 0.6 × 0.5 m (length × width × height) glass cage with a soil substrate (*c.* 120 mm depth). All cages were placed in a room for 2 weeks; temperatures in the room varied from 24 to 32°C with a mean of 28°C, and lights were on a 12: 12 light-dark cycle. Lizards were provided with mealworms (larvae of *Tenebrio molitor*) and water enriched with vitamins and minerals *ad libitum* (Figure 1C).

We then conducted a 2 species (*L. reevesii* and *P. przewalskii*) × 3 temperatures (20, 28, and 36°C) experiment in early August 2020. We chose the three temperatures for the following reasons: 20°C is the lowest nest temperature ever recorded in *L. reevesii* (Lin, 2008), temperatures around 28°C are optimal for egg incubation (embryonic development) in both *L. reevesii* (Lin et al., 2007) and *P. przewalskii* (Wang et al., 2013b), and 36°C is within the range of body temperatures selected by these two species in a thermal gradient (Lin, 2008; Wang et al., 2013a). We equally assigned lizards of each species into one of three temperature treatments. Specifically, we acclimated six individuals of each species to one of three temperatures (20, 28, and 36°C) for 2 weeks, and then collected fecal and small-intestinal samples (Figure 1C). Prior to the experiment, three AAPS (artificial atmospheric phenomena simulator) rooms and all experimental items were disinfected with alcohol wipes and ultraviolet lamps, and lizards were individually weighed to the nearest 0.01 on a Mettler balance and measured for SVL to the nearest 0.1 mm using Mitutoyo digital calipers, and then housed in 1.2 × 0.6 × 0.5 m glass cages. There were 12 lizards (six of each species) in each AAPS room, in which temperature was set at 20 ± 1, 28 ± 1, or 36 ± 1°C and fluorescent lights were switched on at 07:00 h and off at 19:00 h throughout the experiment. Neither in *L. reevesii* nor in *P. przewalskii* did mean values for SVL and body mass differ among the three temperature treatments (one-way ANOVA: *F*_2_,_15_ < 1.808 and *P* > 0.198 in both species). Mealworms sterilized with an ultraviolet lamp 1 h before feeding and distilled water were provided *ad libitum* for 12 days. Feces of each lizard were collected in sterile centrifuge tubes under sterile conditions in the last 2 days of the experiment. We collected small-intestinal contents immediately after collection of fecal samples. All fecal and small-intestinal samples were individually labeled and stored at −80°C for later DNA extraction (Figure 1C).

### DNA extraction and sequencing

Total genomic DNA was extracted from the fecal and small-intestinal samples using the CTAB/SDS method, and concentration and purity were assessed with Nanodrop (Thermo Fisher Scientific, Waltham, MA, USA) and 1.2% agarose gels. The V3–V4 region of the bacterial 16S rRNA was amplified by PCR using the forward primer 338F (5′-ACTCCTACGGGAGGCAGCA-3′) and the reverse primer 806R (5′-GGACTACHVGGGTWTCTAAT-3′) with 7–12 bp unique barcodes. PCR reaction mixture contained 2 μl of DNA template, 5 μl of Q5 reaction buffer (5×), 5 μl of GC buffer (5×), 2 μl of dNTPs (2.5 mM), 1 μl of each primer (10 μM), 0.25 μl of Q5 DNA Polymerase, and 8.75 μl of ddH_2_O. The PCR thermal cycling conditions were set as follows: initial denaturation at 98°C for 2 min, followed by 25 cycles of denaturation at 98°C for 15 s, annealing at 55°C for 30 s, and extension at 72°C for 30 s; and final elongation at 72°C for 5 min. All samples were mixed with an equal molar amount from the purified PCR product of each sample, and library was prepared using the TruSeq Nano DNA LT Library Prep Kit (Illumina, Sangon Biotech Co., Ltd., Shanghai, China). Purified amplicons DNA libraries were sequenced using a MiSeq Reagent Kit V3 (Illumina, Sangon Biotech Co., Ltd., Shanghai, China) on an Illumina MiSeq platform (San Diego, CA, USA) in accordance with Frasergen Bioinformatics (Wuhan, Hubei, China).

### Bioinformatics

We used the Quantitative Insights Into Microbial Ecology 2 (QIIME2 2020.8)^2^ (Caporaso et al., 2010) to process and analyze raw reads, the Cutadapt plugin to trim primers, and the DADA2 plugin to filter reads based on quality, merge paired reads, remove chimeras, and assign reads to amplicon sequence variants (ASVs) (Callahan et al., 2016). We then used the Greengenes 13_8_99% OTUs reference database classifier (DeSantis et al., 2006) to assign the sequence from each ASV, and removed reads identified as the sequences from archaea, chloroplast, and mitochondria. We retained ASVs with more than 100 reads and found in more than two samples, thereby avoiding large partial sample deviations. A phylogenetic tree was constructed using the Mafft alignment algorithm and the Fasttree maximum likelihood estimation (Katoh et al., 2002; Price et al., 2010). To standardize the ASVs abundance information, the sequencing depth was rarefied at 25,000 according to the 95% of the lowest number of sequences of one sample. Alpha and beta diversity metric values were calculated using the “core-metrics-phylogenetic” pipeline through the QIIME2 q2-diversity plugin.

### Data analyses

Four indexes of alpha diversity were chosen, including observed ASVs, Faith’s phylogenetic diversity (Faith’s PD), Shannon index, and Pielou’s evenness index. We used Wilcoxon signed-rank test to examine the differences in these four indexes between the fecal and small-intestinal microbiota, Mann–Whitney test to examine the differences between the two species, and Kruskal–Wallis test to examine the differences among the three temperature treatments.

We calculated three beta diversity distance matrixes, including Bray–Curtis dissimilarity, unweighted UniFrac (which takes the presence and absence of microbial lineages into account, showing the community membership), and weighted UniFrac (which takes the relative abundances of microbial lineages into account, showing the community structure) distances. We used principal coordinates analysis (PCoA) ordinations to visualize sample clustering patterns based on three matrixes. Furthermore, we used the adonis function in vegan package (Oksanen et al., 2017) to perform the permutational multivariate analysis of variance (PERMANOVA, permutation = 9,999) and thereby examine the effects of microbiota source (fecal versus small-intestinal microbiota), host species, temperature treatment, and the interaction between microbiota source and host species on these three distance matrixes. We used the betadisper function in vegan package to perform a beta dispersion test for each matrix and thereby test the homogeneity of dispersion between the fecal and small-intestinal microbiota.

We respectively used Mann–Whitney test and Similarity Percentages (SIMPER) test to identify the phyla and ASVs contributing to the differences in microbial community between the two species. We used a multinomial species classification method (clamtest, clamtest function in vegan) to categorize ASVs into four classes, rare, generalist, warm-climate (*L. reevesii*) specialist, and cold-climate (*P. przewalskii*) specialist. We used linear discriminant analysis (*LDA*) effect size (LEfSe; Segata et al., 2011) to compare the taxa with total relative abundances >1% (from the phylum to genus levels) among the three temperature treatments. Biomarker species in each treatment were detected by Kruskal–Wallis test and linear discriminant analysis.

We compared the differences in bacterial phenotype and gene function among the three temperature treatments and thereby explored the effect of temperature on microbial function. Bacterial phenotypes were inferred through the BugBase web server, including aerobic, anaerobic and facultatively anaerobic bacteria, bacteria containing mobile elements, and biofilm-forming, Gram-negative, Gram-positive, potentially pathogenic, and stress-tolerant bacteria (Ward et al., 2017). Predictive functional profiles of microbial communities were identified by PICRUSt2 based on the Kyoto Encyclopedia of Genes and Genomes (KEGG) database (Douglas et al., 2020). The LEfSe analysis was performed with a threshold criterion of *LDA* > 3 and *P* < 0.05 to compare the relative abundances of function genes from KEGG pathways Level 2 to Level 3 (KEGG orthology, KO) between the two species, and among the three temperature treatments in each species.

Descriptive statistics were expressed as mean ± standard error (SE). All *P-*values were adjusted for false discovery rate using the Benjamini–Hochberg procedure based on the *p*.adjust function in stats package (Bolar, 2019), and the significance level was set at *P* = 0.05.

## Results

### Composition of the fecal and small-intestinal microbiota

We obtained 2,960,930 fecal (36 samples) and 2,963,949 small-intestinal (33 samples) raw reads, from which we further obtained 1,655,845 fecal (mean ± SE: 45,996 ± 2,342) and 1,709,938 (51,816 ± 944) small-intestinal high-quality reads. Of the 33 small-intestinal samples, 17 were collected from *L. reevesii* (6 acclimated to 20°C treatment, 6 acclimated to 28°C, and 5 acclimated to 36°C), and 16 from *P. przewalskii* (6 acclimated to 20°C, 5 acclimated to 28°C, and 5 acclimated to 36°C). The rarefaction curves of estimated ASVs and Shannon index indicated that sequence depths were sufficient to describe of the bacterial community, and that species richness was higher in the small-intestinal microbiota than in the fecal microbiota (Supplementary Figure 1). After normalization, we identified 1,728 ASVs of 14 phyla, 31 classes, 41 orders, 79 families, and 117 genera in the fecal microbiota, and 2,716 ASVs of 20 phyla, 52 classes, 92 orders, 137 families, and 216 genera in the small-intestinal microbiota.

The top four dominant bacterial phyla in the fecal microbiota were Bacteroidetes (41.5% ± 5.5% in *L. reevesii*, and 36.2% ± 4.6% in *P. przewalskii*), Firmicutes (30.3% ± 4.0% in *L. reevesii*, and 36.0% ± 3.9% in *P. przewalskii*), Verrucomicrobia (15.5% ± 4.3% in *L. reevesii*, and 11.7% ± 4.0% in *P. przewalskii*), and Proteobacteria (12.1% ± 3.6% in *L. reevesii*, and 14.8% ± 4.8% in *P. przewalskii*) in both species (Figure 1D and Supplementary Figure 2). The top four dominant fecal bacterial genera were *Akkermansia* (15.4% ± 4.3%), *Bacteroides* (9.8% ± 1.7%), *Desulfovibrio* (7.5% ± 2.5%), and *Parabacteroides* (6.9% ± 1.9%) in *L. reevesii*, and were *Parabacteroides* (13.0% ± 3.9%), *Akkermansia* (11.7% ± 4.0%), *Bacteroides* (7.2% ± 1.3%), and *Oscillospira* (5.8% ± 0.7%) in *P. przewalskii* (Figure 1D and Supplementary Figure 2).

The top four dominant small-intestinal bacterial phyla were Firmicutes (61.7% ± 4.9% in *L. reevesii*, and 41.6% ± 5.1% in *P. przewalskii*), Proteobacteria (38.9% ± 3.6% in *L. reevesii*, and 38.9% ± 3.6% in *P. przewalskii*), Bacteroidetes (17.9% ± 3.2% in *L. reevesii*, and 30.6% ± 5.4% in *P. przewalskii*), and Actinobacteria (9.3% ± 2.0% in *L. reevesii*, and 8.9% ± 2.0% in *P. przewalskii*) in both species (Figure 1E and Supplementary Figure 2). The top four abundant small-intestinal bacterial genera were *Brevibacillus* (7.6% ± 5.4%), *Bacillus* (7.5% ± 4.9%), *Clostridium* (7.1% ± 3.9%), and *Burkholderia* (6.3% ± 1.1%) in *L. reevesii*, and were *Burkholderia* (7.5% ± 1.6%), *Clostridium* (6.5% ± 4.6%), *Bacteroides* (5.7% ± 1.4%), and *Citrobacter* (5.7% ± 5.7%) in *P. przewalskii* (Figure 1E and Supplementary Figure 2).

### Influence of host species on microbial community

**FIGURE 2:**
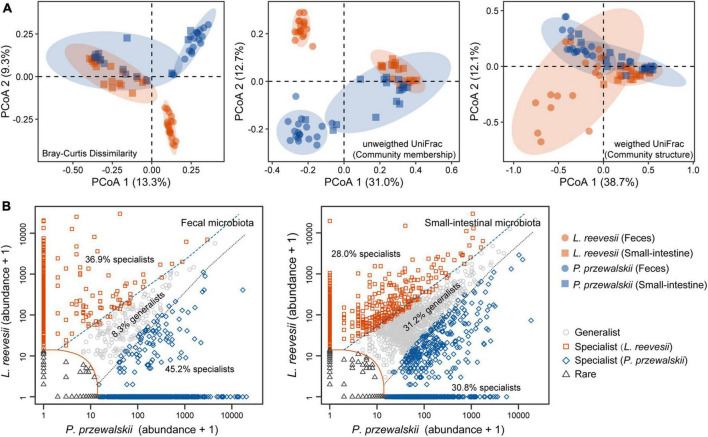
Beta diversity of the fecal and small-intestinal microbiota and distinct microbial taxa classified in *L. reevesii* and *P. przewalskii*. **(A)** Results of principal coordinates analysis (PCoA) based on Bray–Curtis dissimilarity, unweighted UniFrac (community membership) and weighted UniFrac (community structure) distances. **(B)** Four classes categorized by multinomial species classification method (clamtest): for the fecal microbiota, 9.6% were classified as rare, 8.3% as generalist, 36.9% as *L. reevesii* specialist, and 45.2% as *P. przewalskii* specialist; for the small-intestinal microbiota, 10.0% were classed as rare, 31.2% as generalist, 28.0% as *L. reevesii* specialist, and 30.8% as *P. przewalskii* specialist.

Observed ASVs, Faith’s PD, and Shannon indexes revealed that alpha diversity was higher in the small-intestinal microbiota as compared to the fecal microbiota (Wilcoxon signed-rank test, all *P* < 0.03), but evenness index did not show a significant difference in alpha diversity between the small-intestinal and fecal microbiota (Figure 1F; Wilcoxon signed-rank test, *W* = 201, *P* = 0.16). For the fecal microbiota, observed ASVs and Faith’s PD index were greater in *P. przewalskii* than in *L. reevesii* (Mann–Whitney test, both *P* < 0.01), whereas Shannon and evenness indexes did not differ significantly between the two species (Figure 1F; Mann–Whitney test, both *P* > 0.07). For the small-intestinal microbiota, none of the four alpha diversity indexes differed significantly between the two species (Figure 1F; Mann–Whitney test, all *P* > 0.06). Bray–Curtis dissimilarity, unweighted UniFrac, and weighted UniFrac distances revealed that microbiota source (fecal versus small-intestinal microbiota), host species, and their interaction affected beta diversity of microbial communities (Figure 2A and Table 1). Bray–Curtis dissimilarity (*P* < 0.01) and weighted UniFrac (*P* < 0.01) both revealed that distance to group centroid was significantly greater in the fecal than in small-intestinal microbiota (Figure 2A). Host species explained about 16.2% (Bray–Curtis dissimilarity), 29.9% (unweighted UniFrac), or 11.7% (weighted UniFrac) of variation in fecal microbial communities, and about 6.4% (Bray–Curtis dissimilarity), 11.3% (unweighted UniFrac), or 8.2% (weighted UniFrac) of variation in small-intestinal microbial communities (Table 1).

**TABLE 1:** The permutational multivariate analysis of variance (PERMANOVA) of microbiota samples (the fecal and small-intestinal microbiota) based on Bray–Curtis dissimilarity, unweighted UniFrac, and weighted UniFrac distances in two host lizard species (*L. reevesii* and *P. przewalskii*) from thermally different regions.

Variable	Bray–Curtis			Unweighted UniFrac			Weighted UniFrac		
	*R* ^2^	*F*	*P*	*R* ^2^	*F*	*P*	*R* ^2^	*F*	*P*
**All sample**									
Microbiota sample (M)	0.103	8.797	<0.001	0.268	31.388	<0.001	0.237	24.294	<0.001
Host species (H)	0.068	5.805	<0.001	0.097	11.351	<0.001	0.051	5.275	0.003
M × H	0.039	3.339	<0.001	0.056	6.617	<0.001	0.029	2.923	0.023
**Fecal sample**									
Host species (H)	0.162	6.793	<0.001	0.299	15.593	<0.001	0.117	5.273	<0.001
**Small-intestinal sample**									
Host species (H)	0.064	2.272	<0.001	0.113	4.116	<0.001	0.082	2.939	0.008

*P-*values were adjusted for false discovery rate using the Benjamini–Hochberg procedure.

Host-specific microbial taxa resulted in beta diversity divergence between *L. reevesii* and *P. przewalskii*. The relative abundances of the top four dominant phyla were similar between the two species. In both species, Bacteroidetes, Firmicutes, Proteobacteria, and Verrucomicrobia were the top four dominant phyla (with a relative abundance >1%) in the fecal microbiota, and Firmicutes, Proteobacteria, Bacteroidetes, and Actinobacteria were the top four dominant phyla in the small-intestinal microbiota. The relative abundance of Firmicutes was significantly higher in *L. reevesii* than in *P. przewalskii* (Mann–Whitney test, *U* = 63, *P* < 0.01). SIMPER analysis revealed that five dominant fecal ASVs of the phyla Bacteroidetes, Proteobacteria, and Verrucomicrobia explained 13.1% of variation in Bray–Curtis dissimilarity, and that five small-intestinal dominant ASVs of the phyla Firmicutes and Proteobacteria explained 14.1% of variation in Bray–Curtis dissimilarity (Supplementary Table 1). Of the ASVs in the fecal microbiota, 9.6% were classed as rare, 8.3% as generalist, 36.9% as *L. reevesii* specialist, and 45.2% as *P. przewalskii* specialist (Figure 2B). Of the ASVs in the small-intestinal microbiota, 10.0% were classed as rare, 31.2% as generalist, 28.0% as *L. reevesii* specialist, and 30.8% as *P. przewalskii* specialist (Figure 2B). In the fecal microbiota, functional categories associated with carbohydrate metabolism were enriched in *P. przewalskii* (*LDA* = 3.01, *P* < 0.01).

### Influence of acclimation temperature on alpha and beta diversity

Neither in the fecal microbiota nor in the small-intestinal microbiota did the four alpha diversity indexes (observed ASVs, Faith’s PD, Shannon index, and evenness index) differ significantly among *L. reevesii* acclimated to three temperatures (Figure 1F and Supplementary Table 2; Kruskal–Wallis test, all *P* > 0.07). The four alpha diversity indexes in the small-intestinal microbiota also did not differ significantly among *P. przewalskii* acclimated to three temperatures (Figure 1F and Supplementary Table 2; Kruskal–Wallis test, all *P* > 0.06). In the fecal microbiota of *P. przewalskii* we found the following. First, species richness inferred from observed ASVs was highest in the 36°C treatment and lowest in the 20°C treatment, with the 28°C treatment in between (Figure 1F and Supplementary Table 2; Kruskal–Wallis test, *H*_2_,_18_ = 9.07, *P* = 0.02). Second, both Shannon index and evenness index were apparently greater in *P. przewalskii* acclimated to 36°C than in their conspecifics acclimated to 20 and 28°C (Figure 1F and Supplementary Table 2; Kruskal–Wallis test, both *P* < 0.03).

**FIGURE 3:**
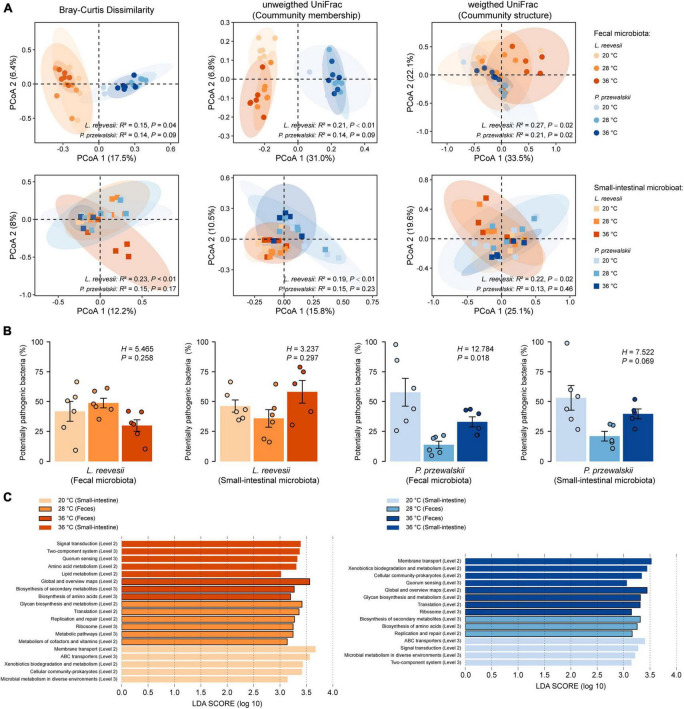
Beta diversity of the fecal and small-intestinal microbiota and the differences in bacterial phenotype and gene function among lizards acclimated to three temperatures (20, 28, and 36°C) for 2 weeks. **(A)** Results of principal coordinates analysis (PCoA) based on Bray–Curtis dissimilarity, unweighted UniFrac and weighted UniFrac distances. **(B)** The relative abundance of potentially pathogenic bacterial (inferred from BugBase) within the fecal and small-intestinal microbiota in *L. reevesii* and *P. przewalskii*. **(C)** KEGG pathway (Level 2 and Level 3) enrichment in lizards acclimated under three thermal conditions (*LDA* > 3 and *P* < 0.05).

Bray–Curtis dissimilarity (feces: *R*^2^ = 0.15, *P* = 0.04; small intestine: *R*^2^ = 0.23, *P* < 0.01), unweighted UniFrac (feces: *R*^2^ = 0.21, *P* < 0.01; small-intestine: *R*^2^ = 0.19, *P* < 0.01), and weighted UniFrac (feces: *R*^2^ = 0.27, *P* = 0.02; small-intestine: *R*^2^ = 0.22, *P* = 0.02) distances revealed that microbial communities differed among *L. reevesii* acclimated to three temperatures (Figure 3A). Weighted UniFrac distance differed among *P. przewalskii* acclimated to three temperatures (Figure 3A; feces: *R*^2^ = 0.21, *P* = 0.02).

### More bacterial taxa are affected by acclimation temperature in *L. reevesii*

The LEfSe results (*LDA* > 3, *P* < 0.05) showed that bacterial taxa were significantly associated with temperature in *L. reevesii*, with two phyla (Bacteroidetes and Firmicutes) and seven genera of the two phyla in the fecal microbiota and one phylum (Proteobacteria) and six genera of the phyla Actinobacteria, Firmicutes, and Proteobacteria in the small-intestinal microbiota varying among the three temperature treatments (Supplementary Figure 3 and Table 2). In *P. przewalskii*, two phyla (Bacteroidetes and Verrucomicrobia) and four genera in the fecal microbiota varied among the three temperature treatments (Supplementary Figure 3 and Table 2). Specifically, the relative abundances of one phylum (Firmicutes) and four genera of the phylum were highest in the 36°C treatment in *L. reevesii*, and the relative abundances of two genera of the phylum Bacteroidetes were highest in the 36°C treatment in *P. przewalskii*. The relative abundance of one phylum (Proteobacteria) and three genera of the phylum were highest in the 20°C treatment and lowest in the 36°C treatment in *L. reevesii* (Table 2). Bacteria of the genera *Clostridium*, *Parabacteroides*, and *Propionibacterium* were enriched in *L. reevesii* acclimated to 28°C, and bacteria of the genera *Akkermansia* and *Clostridium* were enriched in *P. przewalskii* acclimated to the same temperature (Table 2). The relative abundance of the phylum Bacteroidetes increased in *L. reevesii* acclimated to 28°C but decreased in *P. przewalskii* acclimated to the same temperature (Supplementary Figures 3, 4).

**TABLE 2:** Descriptive statistics, expressed as mean ± SE, for the relative abundances of the phyla and genera differing significantly among the three temperature treatments in two host lizard species.

Taxa	% Abundance			Relationship	*LDA*	Group
	20°C	28°C	36°C			
**Phylum**						
Verrucomicrobia	1.41 ± 0.70	**26.86 ± 8.69**	6.89 ± 3.67	↑↓	4.521	*P. przewalskii* (feces)
Bacteroidetes	29.50 ± 9.31	**63.78 ± 6.88**	31.32 ± 5.33	↑↓	4.615	*L. reevesii* (feces)
	42.84 ± 11.06	21.54 ± 4.52	**44.26 ± 2.52**	**↓**↑	4.481	*P. przewalskii* (feces)
Firmicutes	19.50 ± 1.60	26.93 ± 5.56	**44.43 ± 8.11**	↑	4.503	*L. reevesii* (feces)
Proteobacteria	**27.26 ± 4.57**	16.50 ± 5.51	8.37 ± 3.51	↓	4.333	*L. reevesii* (intestine)
**Genus**						
*Clostridium* (p__Firmicutes.f__Clostridiaceae)	0.69 ± 0.20	**18.74 ± 9.96**	0.95 ± 0.32	↑↓	4.317	*L. reevesii* (intestine)
	0.58 ± 0.33	**6.15 ± 3.15**	1.15 ± 0.74	↑↓	3.856	*L. reevesii* (feces)
	0.77 ± 0.39	**3.34 ± 1.97**	0.10 ± 0.07	↑↓	3.626	*P. przewalskii* (feces)
*Akkermansia* (p__Verrucomicrobia)	1.41 ± 0.70	**26.81 ± 8.71**	6.75 ± 3.69	↑↓	4.453	*P. przewalskii* (feces)
*Parabacteroides* (p__Bacteroidetes)	2.04 ± 1.10	**10.08 ± 2.15**	8.47 ± 5.06	↑↓	3.937	*L. reevesii* (feces)
*Propionibacterium* (p__Actinobacteria)	0.42 ± 0.10	**1.18 ± 1.09**	0.09 ± 0.02	↑↓	3.089	*L. reevesii* (intestine)
*Clostridium* (p__Firmicutes.f__Lachnospiraceae)	0.42 ± 0.10	0.02 ± 0.01	**2.27 ± 1.30**	**↓**↑	3.436	*L. reevesii* (feces)
*[Ruminococcus]* (p__Firmicutes)	0.04 ± 0.02	0.03 ± 0.02	**0.25 ± 0.07**	**↓**↑	3.325	*L. reevesii* (feces)
*Magnetospirillum* (p__Proteobacteria)	**1.43 ± 0.33**	0.26 ± 0.11	0.52 ± 0.40	**↓**↑	3.174	*L. reevesii* (intestine)
*Asticcacaulis* (p__Proteobacteria)	**0.79 ± 0.13**	0.20 ± 0.09	0.23 ± 0.08	**↓**↑	3.002	*L. reevesii* (intestine)
*Paludibacter* (p__Bacteroidetes)	0.33 ± 0.20	0.04 ± 0.01	**0.82 ± 0.38**	**↓**↑	3.384	*P. przewalskii* (feces)
*Brevibacillus* (p__Firmicutes)	<0.01	0.09 ± 0.07	**1.49 ± 1.25**	↑	3.201	*L. reevesii* (feces)
*Holdemania* (p__Firmicutes)	0.03 ± 0.01	0.05 ± 0.01	**0.13 ± 0.03**	↑	3.399	*L. reevesii* (feces)
*Rikenella* (p__Bacteroidetes)	0.14 ± 0.08	0.56 ± 0.19	**1.47 ± 0.21**	↑	3.533	*P. przewalskii* (feces)
*Burkholderia* (p__Proteobacteria)	**10.35 ± 1.57**	4.30 ± 1.07	3.79 ± 1.47	↓	3.883	*L. reevesii* (intestine)
*Kocuria* (p__Actinobacteria)	**0.65 ± 0.22**	0.27 ± 0.14	0.08 ± 0.04	↓	3.080	*L. reevesii* (intestine)
*Citrobacter* (p__Proteobacteria)	**2.60 ± 2.16**	0	0	↓	3.424	*L. reevesii* (feces)

The highest abundance of each phylum or genus is in bold.

### Microbial functions are similar between *L. reevesii* and *P. przewalskii*

The relative abundance of potential pathogenic bacteria in the fecal microbiota increased dramatically in *P. przewalskii* acclimated to 20°C (Figure 3B; *H*_2_,_18_ = 12.784, *P* = 0.018). All other functional categories (aerobic, anaerobic and facultatively anaerobic bacteria, bacteria containing mobile elements, and biofilm-forming, Gram-negative, Gram-positive, and stress-tolerant bacteria) did not differ significantly among the three temperature treatments in both species (Supplementary Figure 5; Kruskal–Wallis test, all *P* > 0.05).

The relative abundances of the taxa increased at a given acclimation temperature differed between *L. reevesii* and *P. przewalskii*, but microbial functions enriched at the three acclimation temperatures were similar between the two species. The LEfSe results (*LDA* > 3, *P* < 0.05) showed that the two lizards were similar in the following two aspects. First, microbial pathways involved in global and overview maps, glycan biosynthesis and metabolism, and xenobiotics biodegradation and metabolism differed significantly among the three temperature treatments. Second, five functional categories (KEGG Level 2) associated with cellular processes, environmental information processing, and genetic information processing differed significantly among the three temperature treatments (Figure 3C). More specifically, two functional categories (Global and overview maps, replication and repair) significantly decreased in lizards acclimated to 20°C, and three functional categories [KEGG Level 3, including in metabolism in diverse environments (ko01120), ABC transporters (ko02010) and quorum sensing (ko02024)] significantly increased in lizards acclimated to 20 and 36°C (Figure 3C).

Some differences in the microbial function existed between *L. reevesii* and *P. przewalskii*. Specifically, three functional categories associated with cellular community-prokaryotes, membrane transport, and xenobiotics biodegradation and metabolism were enriched in *L. reevesii* acclimated to 20°C and *P. przewalskii* acclimated to 36°C (Figure 3C). On the contrary, signal transduction was enriched in *L. reevesii* acclimated to 36°C and *P. przewalskii* acclimated to 20°C (Figure 3C). Amino acid metabolism (*LDA* = 3.33, *P* < 0.01) and lipid metabolism (*LDA* = 3.01, *P* < 0.01) was enriched in *L. reevesii* acclimated to 36°C; metabolism of cofactors and vitamins (*LDA* = 3.14, *P* < 0.01) was enriched in *L. reevesii* acclimated to 28°C, but they did not differ among *P. przewalskii* acclimated to the three temperatures (Figure 3C).

## Discussion

Interspecific differences in microbial community were more pronounced in the fecal microbiota than in the small-intestinal microbiota. Bacteroidetes and Firmicutes were the top two dominant phyla in the fecal microbiota and Firmicutes and Proteobacteria were the top two dominant phyla in the small-intestinal microbiota in both species. These findings are consistent with earlier studies on amphibians (Zhou et al., 2020; Zhu et al., 2021), lizards (Ren et al., 2016; Kohl et al., 2017; Zhang et al., 2018; Montoya-Ciriaco et al., 2020; Chen et al., 2022), snakes (Tang W.-J. et al., 2019; Smith et al., 2021; Zhong et al., 2022), and turtles (Qu et al., 2020) where Bacteroidetes, Firmicutes, and Proteobacteria are the top three dominant phyla in the fecal and/or gut microbiota. An earlier study on three sympatric lizard species (*Liolaemus parvus*, *Liolaemus ruibali*, and *Phymaturus williamsi*) shows the stomach, hindgut and fecal microbiota display more pronounced interspecific differences in microbial community than the small-intestinal microbiota (Kohl et al., 2017). We also found that the fecal microbial community was more dissimilar than the small-intestinal microbial community. For example, ASVs of the genera *Akkermansia*, *Butyricicoccus*, and *Cronobacter* were classed as species-specific taxa in the fecal microbial community, but as generalists in the small-intestinal microbial community. More species-specific (*L. reevesii* or *P. przewalskii*) taxa and fewer generalists led to more pronounced interspecific differences in the fecal microbial community than in the small-intestinal microbial community.

The fecal and small-intestinal microbiota could help lizards adapt to thermal environments in which they live, as revealed by the following two pieces of evidence. First, alpha diversity was significantly higher in the cold-climate species (*P. przewalskii*) than in the warm-climate species (*L. reevesii*). High species diversity and functional redundancy are indicators of microbial resilience to return to ecosystem stability after disruption (Sommer et al., 2017). For example, to satisfy the physiological or energetic demands of the host in high-altitude and cold habitats, alpha diversity of the gut microbiota increases with altitude in the plateau pika *O. curzoniae* (Li et al., 2019) and the domestic yak *Bos grunniens* (Fan et al., 2020). Second, functional genes associated with carbohydrate metabolism were enhanced in *P. przewalskii*, conforming to the metabolic cold adaption hypothesis which predicts an increase in the metabolic rate of ectotherms from cold environments compared with their more temperate counterparts (Clarke, 2003; Schaefer and Walters, 2010). In a recent study on two *Phrynocephalus* lizards occurring in thermally different regions, Chen et al. (2022) found that the gut microbiota had a higher abundance of functional categories associated with metabolism in a high-altitude species (*Phrynocephalus erythrurus*) occurring from 4,500 to 5,300 m than in a low-altitude species (*P. przewalskii*) occurring from 500 to 1,700 m. Here, we found that, as in cattle where Firmicutes assimilate nutrients more efficiently in warm tropical environments (Zhang et al., 2022), the relative abundance of Firmicutes in the small-intestinal microbiota was higher in the warm-climate species than in the cold-climate species. Firmicutes are more efficient in dietary caloric intake than Bacteroidetes (Turnbaugh et al., 2006; Krajmalnik-Brown et al., 2012; Semova et al., 2012), and short-chain fatty acids produced by Firmicutes can be directly absorbed by the host as an energy source to promote mass gain (den Besten et al., 2013). That Firmicutes play a more important role in the absorption of nutrients in the warm-climate species further proves the contribution of microbial community to thermal adaptation in lizards.

Environmental temperature alters alpha and beta diversities of the gut microbiota in a diverse array of animal taxa (e.g., Bestion et al., 2017; Fontaine et al., 2018; Zhu et al., 2019, 2021; Cao et al., 2022). Our results showed that the cold-climate species responded to temperature increase or decrease by adjusting alpha diversity of microbial community, whereas the warm-climate species did so by adjusting beta diversity of microbial community. Reduced alpha diversity has been reported for *P. cinereus* (Fontaine et al., 2018), the common lizard *Zootoca vivipara* (Bestion et al., 2017), and the domestic chicken *Gallus domesticus* (Zhu et al., 2019) exposed to warm thermal conditions. Reduced alpha diversity may result in a decrease in genetic diversity and thereby inhibit the host’s ability to respond to environmental stress and resist infection (Pollock et al., 2019). In this study, we found that, as in mice exposed to a high temperature of 36°C (Cao et al., 2022), alpha diversity (observed ASVs and Shannon indices) of the fecal microbiota increased with increasing temperatures in *P. przewalskii*. Such a temperature-dependent change in alpha diversity may be due to the relatively high proportions of *Parabacteroides* and *Citrobacter* in *P. przewalskii* acclimated to 20°C (Figure 1D). The increased relative abundance of some bacterial genera such as *Citrobacter* and *Pseudomonas* of the phylum Proteobacteria reduces microbial diversity in the gut of the brown tree frog *Polypedates megacephalus* artificially hibernating in the laboratory (Weng et al., 2016). In *L. reevesii*, however, we found that neither in the fecal microbiota nor in the small-intestinal microbiota did alpha diversity differ significantly among the three temperature treatments. This finding is consistent with previous studies on the northern leopard frog *Rana pipiens* (Kohl and Yahn, 2016) and the tropical clawed frog *Xenopus tropicalis* (Li et al., 2020). Temperature increase or decrease led to higher microbial heterogeneity in *L. reevesii* than in *P. przewalskii* (Figure 3), indicating that microbial communities were more changeable in the warm-climate species. Plasticity in the gut microbial community potentially helps host animals adapt to harsh environments with extreme climate events, limited food resources, or toxic exposure (Alberdi et al., 2016). The responses of microbial community to temperature increase or decrease differed between the two agamid lizards, and this difference presumably results from the fact that they use thermally different habitats and differ in thermal tolerance (Lin, 2008; Qu et al., 2011; Wang, 2013).

The microbial composition was thermally more sensitive in *L. reevesii* than in *P. przewalskii*. Fecal or small-intestinal bacteria of the phyla Bacteroidetes, Firmicutes, and Proteobacteria and 12 genera belonging to the phyla Actinobacteria, Bacteroidetes, Firmicutes, and Proteobacteria were affected by acclimation temperature in *L. reevesii*. However, in *P. przewalskii*, only fecal bacteria of the phyla Bacteroidetes and Verrucomicrobia and four genera belonging to the phyla Bacteroidetes, Firmicutes, and Verrucomicrobia were affected by acclimation temperature. The trends of variation in the relative abundance of Bacteroidetes and Verrucomicrobia differed between the two lizard species. More specifically, bacteria of the phylum Bacteroidetes enriched in the 20 and 36°C treatments in *P. przewalskii*, bacteria of the phylum Bacteroidetes enriched in the 28°C treatment in *L. reevesii*, and bacteria of the phylum Verrucomicrobia enriched in the 28°C treatment in *P. przewalskii*. Bacteria of the phyla Bacteroidetes and Verrucomicrobia can use host-derived mucin glycans in the absence of dietary substrates (Sonnenburg et al., 2005; Carey et al., 2013). It has been reported for *R. dybowskii* (Tong et al., 2020), *I. tridecemlineatus* (Dill-McFarland et al., 2014; Stevenson et al., 2014), and *U. arctos* (Sommer et al., 2016) that the increased relative abundance of Bacteroidetes or Verrucomicrobia and the decreased relative abundance of Firmicutes during hibernation help hosts adapt to long-term fasting in winter. The increased relative abundance of Verrucomicrobia was observed in *L. reevesii* acclimated to 20°C, though statistically not significant. Additionally, as in *Andrias davidianus* (Zhu et al., 2021), the relative abundance of Firmicutes and Proteobacteria increased at low or high acclimation temperatures in *L. reevesii*. In *Drosophila subobscura*, decisive effects of heat stress on the gut microbiota composition differ between the sexes, with warming reducing the relative abundances of Firmicutes in females, increasing the relative abundances of Firmicutes, and reducing the relative abundances of Proteobacteria in males (Jaramillo and Castañeda, 2021). The result for male *D. subobscura* is consistent with this study based on male adults of *L. reevesii*. High Firmicutes abundance (or Firmicutes/Bacteroidetes ratio) is related to obesity in humans (Clemente et al., 2012), and to weight gain and immune function in birds (Zhang et al., 2015) and mammals (Ley et al., 2005). The Firmicutes/Bacteroidetes ratio increased with elevated temperature in *L. reevesii*, indicating an increased capacity for energy harvest. However, in cold-climate vertebrates such as the Brandt’s vole *Lasiopodomys brandtii* (Bo et al., 2019) and the Tibetan sheep *Ovis aries* (Fan et al., 2021), cold stress increases the Firmicutes/Bacteroidetes ratio to maintain the host’s metabolic balance. Compared with active counterparts, hibernating animals often have a higher relative abundance of Proteobacteria in their gut microbiota (Weng et al., 2016; Song et al., 2020; Tong et al., 2020; Cao et al., 2023). Such a difference is also evident in animals not hibernating in winter (Sun et al., 2016; Ferguson et al., 2018), indicating that the bacterial phylum Proteobacteria may have an important role in helping hosts adapt to fasting (starvation) or cold conditions. It is worth noting that most taxa of the phylum Proteobacteria have pathogenic or antipathogenic functions (Wiggins et al., 2011; Ramsey et al., 2015). The relative abundances of putative pathogens did not differ significantly among three temperature treatments in *L. reevesii*, but were enriched in *P. przewalskii* acclimated to 20°C. The accelerated colonization of pathogenic bacteria in the gut at low temperatures has been observed in other cold-climate vertebrates (Liu et al., 2022; Tong et al., 2023). Bacteria of the genus *Akkermansia* can enhance metabolic homeostasis and anti-inflammatory properties (Everard et al., 2013; Shin et al., 2014). The relative abundance of *Akkermansia* bacteria decreased in *P. przewalskii* acclimated to 20°C, suggesting that, as in *X. tropicalis* (Li et al., 2020), low-temperatures exposure may lead to a decrease in immunity in this cold-climate lizard. Temperature-dependent changes in the microbial composition differed between *L. reevesii* and *P. przewalskii*, and this difference can be explained by the following two reasons. First, host genetic background is a key determinant of the microbial composition and structure (Kokou et al., 2018; Weinstein et al., 2021). Second, host thermal tolerance modulates the microbial response to temperature change (Kokou et al., 2018). Overall, our data showed that the microbial community was thermally more sensitive in the warm-climate lizard than in the cold-climate lizard, and that lizards can adapt to temperature increase or decrease by adjusting the gut microbiota.

Although the influence of acclimation temperature on the microbial composition and structure differed between *L. reevesii* and *P. przewalskii*, the two species exhibited similar microbial functions enriched at different acclimation temperatures. Pathways related to environmental information processing (i.e., ABC transporters and two-component system) were enriched in lizards acclimated to 20 or 36°C, confirming the existence of a functional response of the gut microbial community to temperature (Nachin et al., 2005). ABC transporters pathway uses ATP hydrolysis to transport a wide variety of substrates (i.e., sugars, amino acids, glycans, and phospholipids) (Gottesman and Ambudkar, 2001). This pathway might promote food absorption to help the host survive in the season or environment that is cold and/or short of food (Xia et al., 2021). Two-component system pathway is involved in the information communication between microbes and their environment, allowing bacteria to sense and respond to environmental change. Consistent with a study on goitred gazelles (*Gazella subgutturosa*) exposed to low temperatures in winter (Qin et al., 2022), our study showed that the relative abundant of two-component system was significantly higher in *P. przewalskii* acclimated to the lowest temperature (20°C). This finding suggests the role of two-component system in cold adaptation. Additionally, the temperature-dependence of pathways associated with amino acid metabolism, lipid metabolism, and metabolism of cofactors and vitamins seen in *L. reevesii* suggests that metabolic functions of bacteria are thermally more sensitive in warm-climate lizards. Consistent with studies on the Wistar rat *Rattus norvegicus* (Qu et al., 2021) and the rice frog *Fejervarya limnocharis* (Huang and Liao, 2021), amino acid metabolism and lipid metabolism were enriched in *L. reevesii* acclimated to 36°C. Overall, both species of lizards studied herein, *L. reevesii* in particular, could cope with temperature changes by adjusting the relative abundance of functional categories associated with metabolism and environmental information processing.

## Conclusion

With the findings of this study, we can draw the following conclusions. First, the fecal microbiota displays more pronounced interspecific differences in microbial community than the small-intestinal microbiota in both species of lizards occurring in thermally different regions. Second, the response of fecal and small-intestinal microbiota to temperature increase or decrease differs between the warm-climate (*L. reevesii*) and cold-climate (*P. przewalskii*) species, with more bacterial taxa affected by acclimation temperature in *L. reevesii* than in *P. przewalskii.* Third, both species, the warm-climate species in particular, can cope with temperature changes by adjusting the relative abundance of functional categories associated with metabolism and environmental information processing. Fourth, the fecal microbiota contributes to cold-climate adaptation in *P. przewalskii*, as revealed by the fact that functional genes associated with carbohydrate metabolism were enhanced in this species. Taken together, our results validate the hypotheses tested, of which one suggests that the gut microbiota should help lizards adapt to thermal environments in which they live and one suggests that microbial communities should be thermally more sensitive in warm-climate lizards than in cold-climate lizards.

## Data availability statement

The datasets presented in this study can be found in online repositories. The names of the repository/repositories and accession number(s) can be found at: https://www.ncbi.nlm.nih.gov/, PRJNA901369.

## Ethics statement

The animal study was approved by the Laboratory Animal Care and Animal Ethics Committee of Nanjing Normal University and Hainan Tropical Ocean University. The study was conducted in accordance with the local legislation and institutional requirements.

## Author contributions

X-MZ: Data curation, Formal analysis, Investigation, Methodology, Validation, Visualization, Writing – original draft, Writing – review & editing. J-QC: Investigation, Writing – review & editing. YD: Funding acquisition, Investigation, Writing – review & editing. C-XL: Investigation, Resources, Writing – review & editing. Y-FQ: Data curation, Formal analysis, Writing – review & editing. L-HL: Conceptualization, Funding acquisition, Investigation, Supervision, Writing – original draft, Writing – review & editing. XJ: Conceptualization, Funding acquisition, Investigation, Project administration, Resources, Supervision, Writing – original draft, Writing – review & editing.

## Footnotes

1
https://data.cma.cn/

2
https://qiime2.org/

## Supplementary material

Supplementary Figure 1 Rarefaction curves based on observed ASVs **(A)** and Shannon index **(B)** for each sample.

Supplementary Figure 2 The relative abundance of the fecal **(A)** and small-intestinal **(B)** microbiota at the phylum and genus levels in *L. reevesii* and *P. przewalskii*. LC, LM, and LH represent *L. reevesii* acclimated to 20, 28, and 36°C, respectively; PC, PM, and PH represent *P. przewalskii* acclimated to 20, 28, and 36°C, respectively.

Supplementary Figure 3 Biomarker taxa of the fecal and small-intestinal microbiota identified by LEfSe in lizards acclimated under three thermal conditions. **(A)** Fecal microbiota of *L. reevesii*; **(B)** small-intestinal microbiota of *L. reevesii*; **(C)** fecal microbiota of *P. przewalskii*; **(D)** small-intestinal microbiota of *P. przewalskii*. Letters p, c, o, f, and g represent phylum, class, order, family, and genus, respectively.

Supplementary Figure 4 The relative abundance of key bacteria in lizards acclimated under three thermal conditions.

Supplementary Figure 5 The relative abundance of potential microbial phenotypes (inferred from BugBase, except potentially pathogenic bacteria) within the fecal **(A)** and small-intestinal **(B)** microbiota in *L. reevesii* and *P. przewalskii*.
