# Supplementary figures and images for: Microbial communities are thermally more sensitive in warm-climate lizards compared with their cold-climate counterparts
Source: Front Microbiol. 2024 Apr 15;15:1374209. doi: 10.3389/fmicb.2024.1374209 (PMC11056556; doi:10.3389/fmicb.2024.1374209)

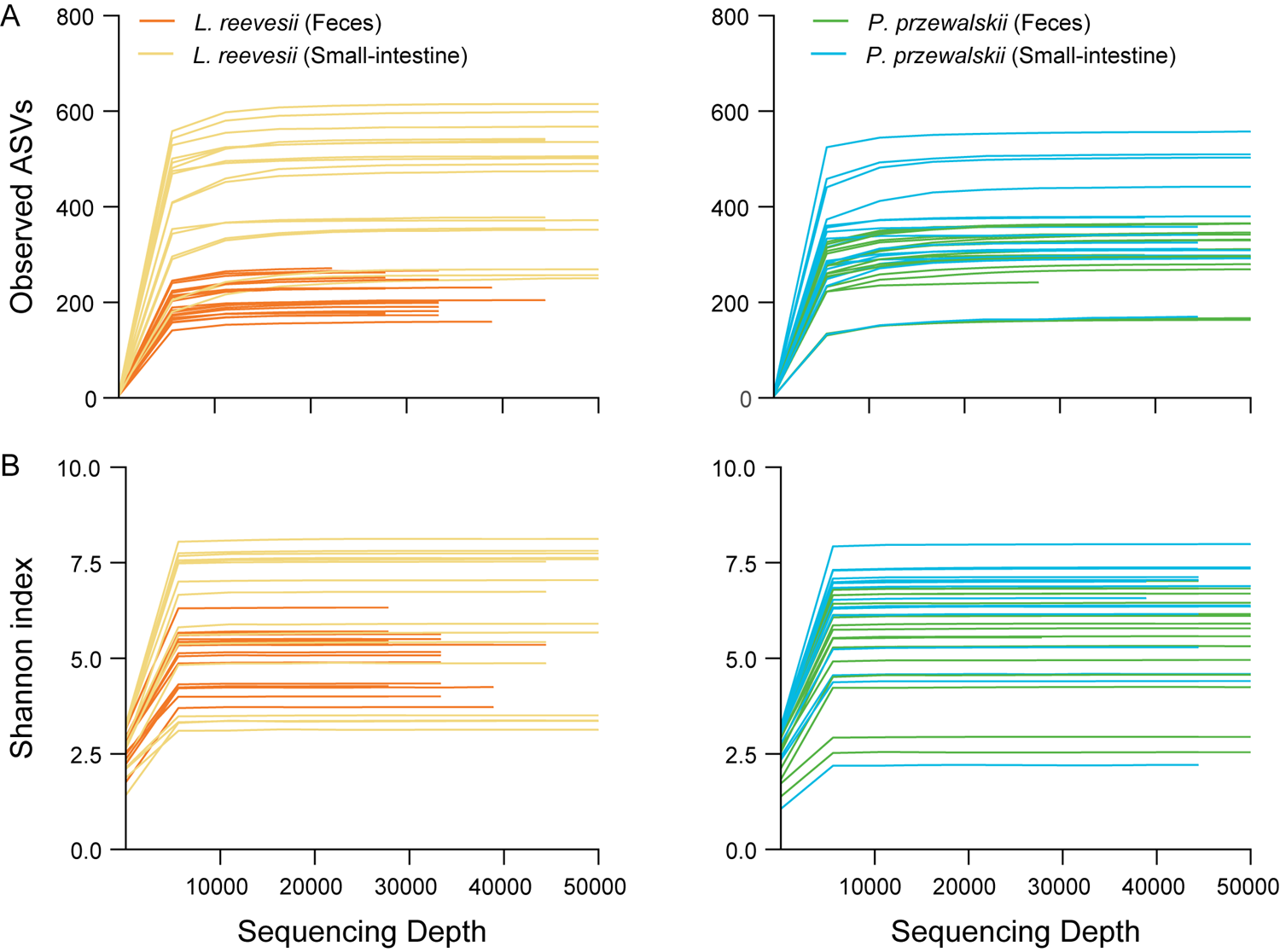

Supplement: Supplementary Figure 1 — Rarefaction curves based on observed ASVs (A) and Shannon index (B) for each sample. [file Image_1.TIF]

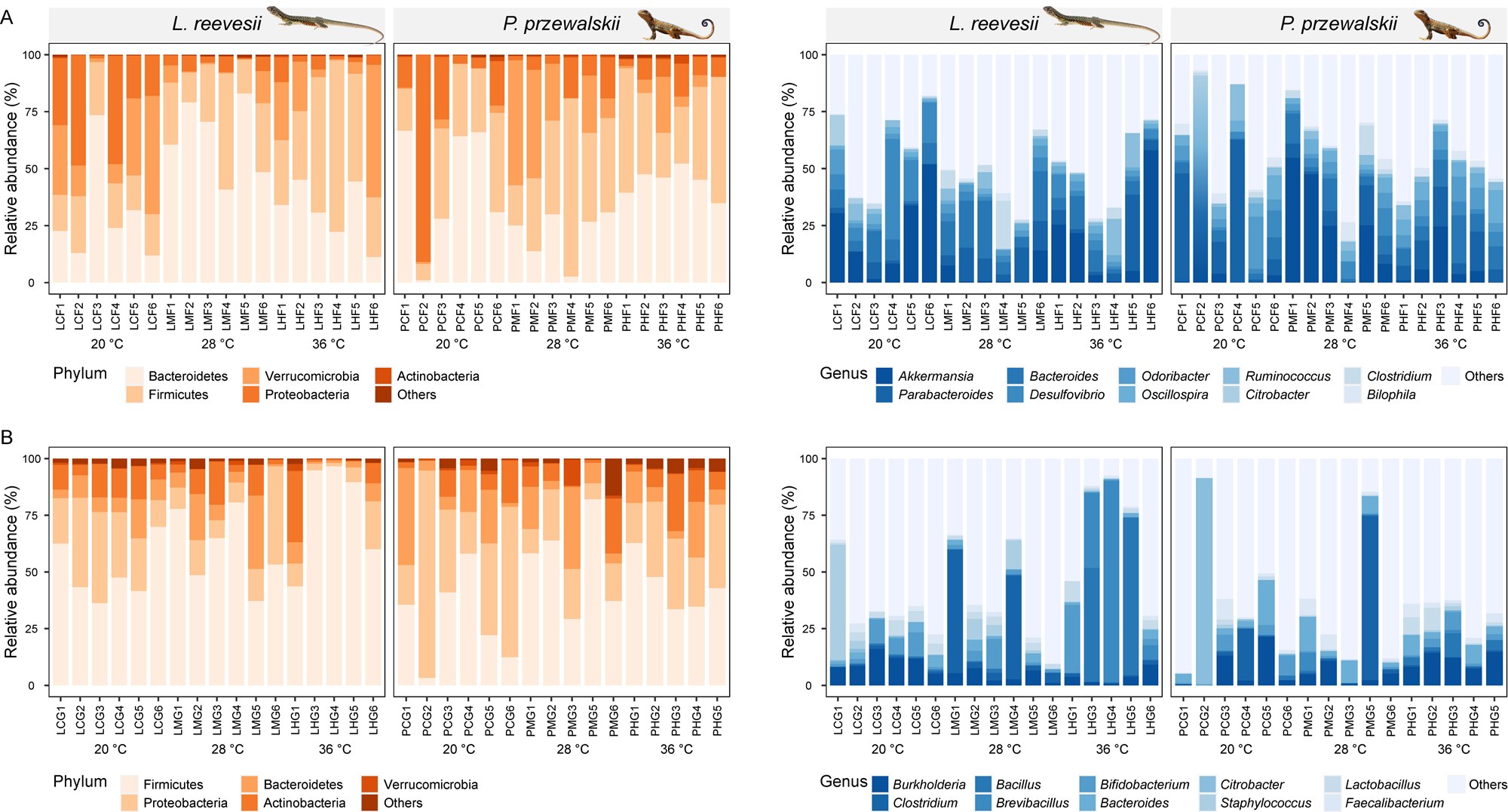

Supplement: Supplementary Figure 2 — The relative abundance of the fecal (A) and small-intestinal (B) microbiota at the phylum and genus levels in L. reevesii and P. przewalskii. LC, LM, and LH represent L. reevesii acclimated to 20, 28, and 36°C, respectively; PC, PM, and PH represent P. przewalskii acclimated to 20, 28, and 36°C, respectively. [file Image_2.TIF]

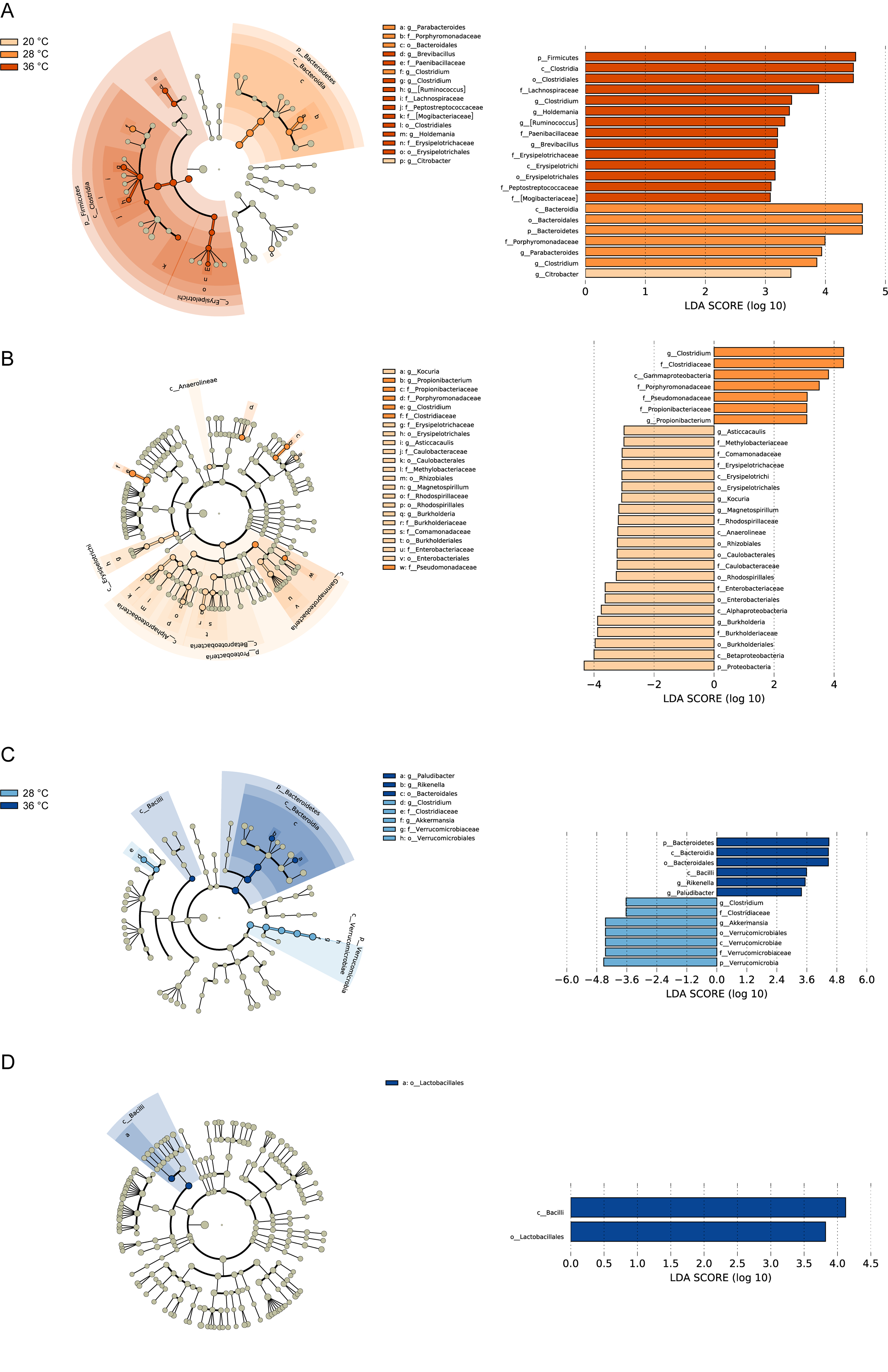

Supplement: Supplementary Figure 3 — Biomarker taxa of the fecal and small-intestinal microbiota identified by LEfSe in lizards acclimated under three thermal conditions. (A) Fecal microbiota of L. reevesii; (B) small-intestinal microbiota of L. reevesii; (C) fecal microbiota of P. przewalskii; (D) small-intestinal microbiota of P. przewalskii. Letters p, c, o, f, and g represent phylum, class, order, family, and genus, respectively. [file Image_3.TIF]

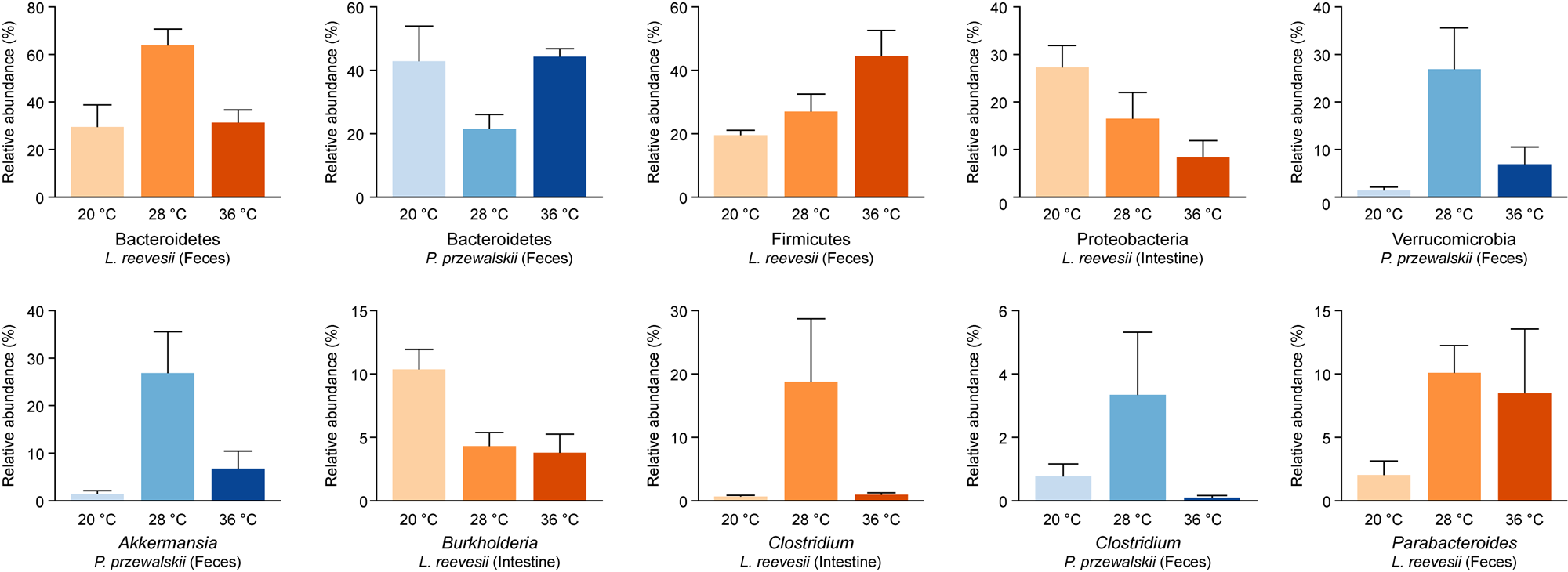

Supplement: Supplementary Figure 4 — The relative abundance of key bacteria in lizards acclimated under three thermal conditions. [file Image_4.TIF]

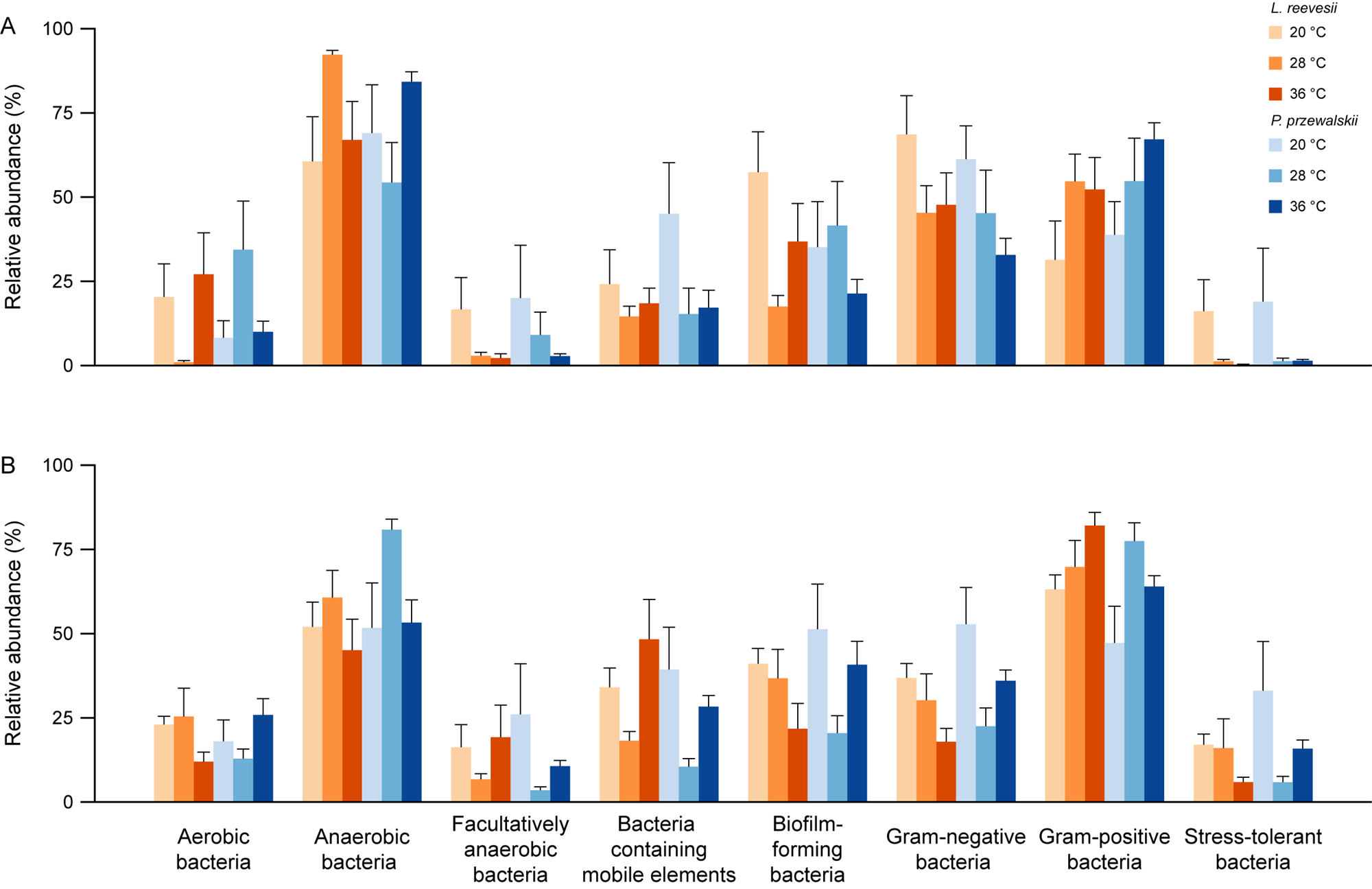

Supplement: Supplementary Figure 5 — The relative abundance of potential microbial phenotypes (inferred from BugBase, except potentially pathogenic bacteria) within the fecal (A) and small-intestinal (B) microbiota in L. reevesii and P. przewalskii. [file Image_5.TIF]
