# Supplementary material for: Microbial communities are thermally more sensitive in warm-climate lizards compared with their cold-climate counterparts
Source: Front Microbiol. 2024 Apr 15;15:1374209. doi: 10.3389/fmicb.2024.1374209 (PMC11056556; doi:10.3389/fmicb.2024.1374209)
Supplement: Supplementary file 6 [file Table_1.DOCX]

**TABLE S1** SIMPER analysis of five dominant ASVs that contribute to Bray-Curtis dissimilarity between two agamid lizards.

| ASV | Taxonomic classification | Relative abundance % | | SIMPER percentage % | Contribution  % |
| --- | --- | --- | --- | --- | --- |
|  |  | *L. reevesii* | *P. przewalskii* |  |  |
| **Fecal samples** | | | | | |
| ASV1445 | Verrucomicrobia | 6.654 | 0.009 | 3.328 | 3.388 |
| ASV1114 | Verrucomicrobia | 5.579 | 0.001 | 2.789 | 2.839 |
| ASV785 | Bacteroidetes | 4.547 | 0.000 | 2.274 | 2.315 |
| ASV2014 | Verrucomicrobia | 0.000 | 4.529 | 2.265 | 2.305 |
| ASV638 | Proteobacteria | 4.363 | 0.000 | 2.181 | 2.221 |
| **Small-intestinal samples** | | | | | |
| ASV1749 | Firmicutes | 6.916 | 0.269 | 3.479 | 3.986 |
| ASV2725 | Proteobacteria | 0.017 | 4.633 | 2.322 | 2.661 |
| ASV1128 | Firmicutes | 4.590 | 0.000 | 2.295 | 2.629 |
| ASV2652 | Proteobacteria | 4.940 | 5.702 | 2.144 | 2.457 |
| ASV2118 | Firmicutes | 2.948 | 1.658 | 2.063 | 2.364 |
