# Supplementary material for: Microbial communities are thermally more sensitive in warm-climate lizards compared with their cold-climate counterparts
Source: Front Microbiol. 2024 Apr 15;15:1374209. doi: 10.3389/fmicb.2024.1374209 (PMC11056556; doi:10.3389/fmicb.2024.1374209)
Supplement: Supplementary file 7 [file Table_2.DOCX]

**TABLE S2** Alpha diversity of fecal and small-intestinal microbiota and results of Kruskal-Wallis *H* test with adjusted *P* value using the Benjamini-Hochberg procedure in lizards acclimated under three thermal conditions. Data expressed as mean ± SE and range

| Indexes | 20 °C | 28 °C | 36 °C | Statistical results |
| --- | --- | --- | --- | --- |
| ***L. reevesii*, fecal microbiota** | | | | |
| Observed ASV | 183.33 ± 6.85  (158-201) | 217.83 ± 14.04  (172-251) | 238.00 ± 13.38  (200-271) | *H* = 6.823, *P* = 0.033, adj *P* = 0.071 |
| Faith’s PD | 24.17 ± 1.25  (18.55-27.58) | 27.01 ± 0.89  (23.51-29.45) | 29.34 ± 1.56  (25.17-34.48) | *H* = 5.298, *P* = 0.071, adj *P* = 0.071 |
| Shannon | 4.57 ± 0.26  (3.72-5.35) | 5.35 ± 0.22  (4.28-5.70) | 5.39 ± 0.28  (4.24-6.33) | *H* = 6.421, *P* = 0.040, adj *P* = 0.071 |
| Evenness | 0.61 ± 0.03  (0.51-0.70) | 0.69 ± 0.02  (0.58-0.73) | 0.68 ± 0.03  (0.54-0.79) | *H* = 5.836, *P* = 0.054, adj *P* = 0.071 |
| ***L. reevesii*, small-intestinal microbiota** | | | | |
| Observed ASV | 493.17 ± 40.83  (371-613) | 450.50 ± 49.39  (266-595) | 376.40 ± 61.67  (239-537) | *H* = 2.430, *P* = 0.297, adj *P* = 0.297 |
| Faith’s PD | 48.73 ± 2.28  (40.86-53.52) | 52.40 ± 3.21  (40.94-61.07) | 43.26 ± 4.34  (34.10-57.64) | *H* = 3.681, *P* = 0.159, adj *P* = 0.212 |
| Shannon | 7.19 ± 0.49  (4.87-8.12) | 5.93 ± 0.64  (3.38-7.62) | 4.86 ± 0.96  (3.13-7.62) | *H* = 4.882, *P* = 0.087, adj *P* = 0.174 |
| Evenness | 0.80 ± 0.05  (0.57-0.88) | 0.67 ± 0.06  (0.42-0.83) | 0.56 ± 0.10  (0.39-0.85) | *H* = 5.248, *P* = 0.072, adj *P* = 0.174 |
| ***P. przewalskii*, fecal microbiota** | | | | |
| Observed ASV | 246.17 ± 28.70 (159-323) | 298.83 ± 14.42  (264-364) | 344.83 ± 5.50  (328-362) | ***H* = 9.074, *P* = 0.011, adj *P* = 0.022** |
| Faith’s PD | 29.09 ± 2.74 (21.06-37.41) | 32.48 ± 1.41  (28.72-37.88) | 35.50 ± 0.57  (33.76-37.53) | *H* = 4.363, *P* = 0.113, adj *P* = 0.113 |
| Shannon | 4.94 ± 0.71  (2.56-6.69) | 5.13 ± 0.28  (4.23-6.13) | 6.62 ± 0.17  (5.89-7.02) | ***H* = 9.088, *P* = 0.011, adj *P* = 0.022** |
| Evenness | 0.62 ± 0.08  (0.35-0.81) | 0.62 ± 0.03  (0.52-0.72) | 0.79 ± 0.02  (0.70-0.83) | ***H* = 7.450, *P* = 0.024, adj *P* = 0.032** |
| ***P. przewalskii*, small-intestinal microbiota** | | | | |
| Observed ASV | 322.00 ± 38.72  (166-441) | 346.00 ± 38.59  (291-499) | 426.60 ± 43.12  (340-554) | *H* = 3.503, *P* = 0.173, adj *P* = 0.203 |
| Faith’s PD | 38.17 ± 3.45  (22.68-47.52) | 41.02 ± 1.49  (38.34-46.60) | 45.17 ± 2.40  (39.37-51.92) | *H* = 3.188, *P* = 0.203, adj *P* = 0.203 |
| Shannon | 5.49 ± 0.73  (2.20-6.86) | 5.91 ± 0.50  (4.41-7.36) | 7.30 ± 0.18  (7.02-7.97) | *H* = 7.165, *P* = 0.028, adj *P* = 0.056 |
| Evenness | 0.66 ± 0.08  (0.30-0.80) | 0.70 ± 0.05  (0.54-0.82) | 0.84 ± 0.01  (0.82-0.87) | *H* = 8.416, *P* = 0.015, adj *P* = 0.056 |
